# Unknown HIV status and the TB/HIV collaborative control program in Ethiopia: systematic review and meta-analysis

**DOI:** 10.1186/s12889-020-09117-2

**Published:** 2020-06-29

**Authors:** Balew Arega, Abraham Minda, Getachew Mengistu, Mulugeta Endale, Asnake Agunie

**Affiliations:** 1 Yekatit 12 Hospital Medical College, P.O. Box. 257, Addis Ababa, Ethiopia; 2 Debere Markos University, College of Health Sciences, P.O. Box,269, Debere Markos, Ethiopia; 3 Addis Ababa City Administrative Health Bureau, Addis Ababa, Ethiopia

**Keywords:** Unknown HIV status, Tuberculosis, Ethiopia

## Abstract

**Background:**

Ethiopia has shown significant efforts to address the burden of TB/HIV comorbidity through the TB/HIV collaborative program. However, these diseases are still the highest cause of death in the country. Therefore, this systematic review and meta-analysis evaluated this program by investigating the overall proportion of unknown HIV status among TB patients using published studies in Ethiopia.

**Methods:**

We conducted a systematic review and meta-analysis of published studies in Ethiopia. We identified the original studies using the databases MEDLINE/PubMed, and Google Scholar. The heterogeneity across studies was assessed using Cochran’s Q test and I ^2^ statistics. The Begg’s rank correlation and the Egger weighted regression tests were assessed for the publication bias. We estimated the pooled proportion of unknown HIV status among TB patients using the random-effects model.

**Results:**

Overall, we included 47 studies with 347,896 TB patients eligible for HIV test. The pooled proportion of unknown HIV status among TB patients was 27%(95% CI; 21–34%) and with a substantial heterogeneity (I^2^ = 99.9%). In the subgroup analysis, the pooled proportion of unknown HIV status was 39% (95% CI; 25–54%) among children and 20% (95% CI; 11–30%) among adults. In the region based analysis, the highest pooled proportion of unknown HIV status was in Gambella, 38% (95% CI; 16–60%) followed by Addis Ababa, 34%(95% CI; 12–55%), Amhara,30%(95% CI; 21–40%),and Oromia, 23%(95% CI; 9–38%). Regarding the study facilities, the pooled proportion of unknown HIV status was 33% (95% CI; 23–43%) in the health centers and 26%(95% CI; 17–35%) in the hospitals. We could not identify the high heterogeneity observed in this review and readers should interpret the results of the pooled proportion analysis with caution.

**Conclusion:**

In Ethiopia, about one-third of tuberculosis patients had unknown HIV status. This showed a gap to achieve the currently implemented 90–90-90 HIV/AIDS strategic plan in Ethiopia, by 2020. Therefore, Ethiopia should strengthen TB/HIV collaborative activities to mitigate the double burden of diseases.

## Background

Human immunodeficiency virus (HIV) and tuberculosis (TB) co-infection represent a significant cause of morbidity and mortality. This is in part due to the shared nature of immune defense against the two diseases [1, 2]. Low-income countries, the African continent with 74% new TB/HIV co-infection rate, take a great share of TB/HIV cases worldwide [3].

The World Health Organization (WHO), recognizing the synergistic effect of these diseases, recommended a framework of TB/HIV collaborative strategic program carried out across the health facilities [4]. These include routine testing of all TB patients for HIV, symptomatic TB screening of HIV patients, and early initiation of prophylaxis and treatment [5, 6]. Many countries implemented this program record a significant improvement, but less than half of all TB patients tested for HIV infection worldwide [7].

Ethiopia adopted this global recommendation early and embarked on the service (by establishing HIV/TB Advisory Committee) since 2002. The program expanded across the health facilities for the last one and half decades [8, 9]. A recent national report, however, found that up to 19% of TB patients did not know their HIV status, and a significant variation (0–24%) was observed across the regions [10]. The success, sustainability and coverage of this program, also the millennium development goal completed in 2015 [11], has been challenged. The problem might be related to the programs multifaceted nature with the sharing project mode approaches and their funding dependent on HIV-related indicators [12].

Currently, the country implemented the 90–90-90 HIV/AIDS global strategy that calls for 90% of HIV-infected individuals to be diagnosed by 2020, 90% of whom will be on antiretroviral therapy (ART) and 90% of whom will attain sustained biological suppression [13]. However, the trend of HIV/AIDS for the last 26(1990–2016) years and predicting achievement of the 90–90-90 HIV prevention targets found that, Ethiopia is not in a good position to achieve the first target for HIV diagnosis [14, 15].

Unknown HIV status among tuberculosis patients could be a potential source of the ongoing increase in the disease. It also destabilizes HIV control programs practiced throughout the country. To our knowledge, there is no comprehensive published data assessing the unknown HIV status among TB patients since the TB/HIV program has been implemented in Ethiopia. Therefore, this systematic review and meta-analysis determined the magnitude of unknown HIV status among TB patients. It also assessed variation of unknown HIV status across the regions and health settings in the country. For this purpose, we reviewed published studies conducted using TB/HIV routine data in Ethiopia.

## Methods

### Study design and data sources

We conducted a systematic review and meta-analysis of published studies in Ethiopia to estimate the proportion of unknown HIV status among TB patients after the HIV/TB collaborative approach was started. For this, we searched original articles using MEDLINE/PubMed, Embase, Cochrane library, and Google scholar databases. Besides, we made a hand search for cross-referencing the identified original articles. To report the results of the current meta-analysis, we used the Preferred Reporting Items for Systematic reviews and Meta-Analysis (PRISMA) guidelines [16]. The electronic search was performed using a combination of keywords in the MEDLINE/PubMed database using the Medical Science Heading (MeSH) terms [(Tuberculosis OR TB [MeSH Terms)] AND (HIV OR AIDS [MeSH Terms] OR HIV/AIDS [MeSH Terms] OR ‘Human immunodeficiency virus’ [MeSH Terms] OR ‘Human Acquired immunodeficiency syndrome’[MeSH Terms]) AND (Retrospective) AND (Ethiopia)]. We included articles published in English language and among humans. The review comprised studies conducted between 2002 and 2019. We did the last search on 30 September 2019.

### Study selection

In the first stage, we reviewed the titles and abstracts of all retrieved articles addressing the study questions and grouped them as eligible. In the second stage, we evaluated each article in detail against the inclusion criteria. The inclusion criteria were:-a published study in Ethiopian after 2002, retrospective studies using routine data, reported quality control/assurance measures, and report the HIV status of TB patients. We excluded studies with a prospective study design (collected data for research purpose), done among known HIV patients, and reviews that reiterated findings from the already included studies. Two authors (BA and GM) conducted the articles selection and any disagreement resolved through discussion.

### Study quality assessment

We tested the quality of the included studies using the Joanna Briggs Institute (JBI) appraisal tool for prevalence studies [17]. The tool has nine appraisal criteria and for each criterion,'Yes’, ‘No’, or ‘Unclear’ were likely responses. An article fulfilled the evaluation criteria ‘yes’ answer received 1 point; otherwise, it scored 0 points. After the evaluation, we included articles with a high-quality score that fulfilled more than half of the evaluation JBI criteria.

### Data extraction

With the help of a standardized data abstraction format prepared in Microsoft Excel, two authors (BA and GM) extracted important data related to study characteristics. This includes the title, first author, publication year, year of study, design of the study, regions of study (study site in the country), HIV status (positive, negative, or unknown), study settings (hospitals, health centers), the total number of tuberculosis cases, types of tuberculosis (Acid fast bacilli (AFB)-positive, AFB-negative, extrapulmonary cases (EPTB)), the data type, and age groups of patients. The authors (BA and GM) extracting the data, solved any disagreement that happened through discussion and consensus.

### Statistical analysis

We extracted the data and analysed using Stat version 14 software. Then, we presented a detailed description of the original studies in a table and forest plot. We determined the pooled estimate of the proportion of unknown HIV status using the DersimonianLaird for random-effects meta-analysis (random effects model). The proportion of unknown HIV status among tuberculosis was measured with 95% confidence intervals (CIs). We used Arcsine transformation and presented the results on the original probability scale after using the corresponding back-transformation [18].

We tested the potential source of publication bias and heterogeneity across studies using the Cochrane Q test (presence of heterogeneity) and I^2^ statistics (amount of heterogeneity). To check the presence of heterogeneity, we used the Cochrane Q test and significant heterogeneity taken when *P* < 0.10. The I^2^ was used to measure the level of heterogeneity between studies with the values of 25, 50, and 75% which is to mean low medium, and high heterogeneity, respectively [19]. We also used the Begg’s rank correlation test and Egger weighted regression tests to determine the publication bias. A significant publication bias considered if *p* < 0.05.

We did a sensitivity test to indicate which study is the prime determinant of the pooled result, and the principal source of heterogeneity. The test exclude each study one by one in the analysis to show the change in pooled effect size and associated heterogeneity. If the point estimate of pooled prevalence after dropping a study lies within the 95% CI of the overall pooled estimate for all studies combined, we considered the study has a non-important influence on the overall pooled estimate [20].

We planned subgroup analysis at the point of designing the study to investigate the source of heterogeneity based on administrative regions, study setting, age groups, and TB types. The minimum number of studies should be at least two in each subgroup analysis.

## Results

### Search results

**Fig. 1:**
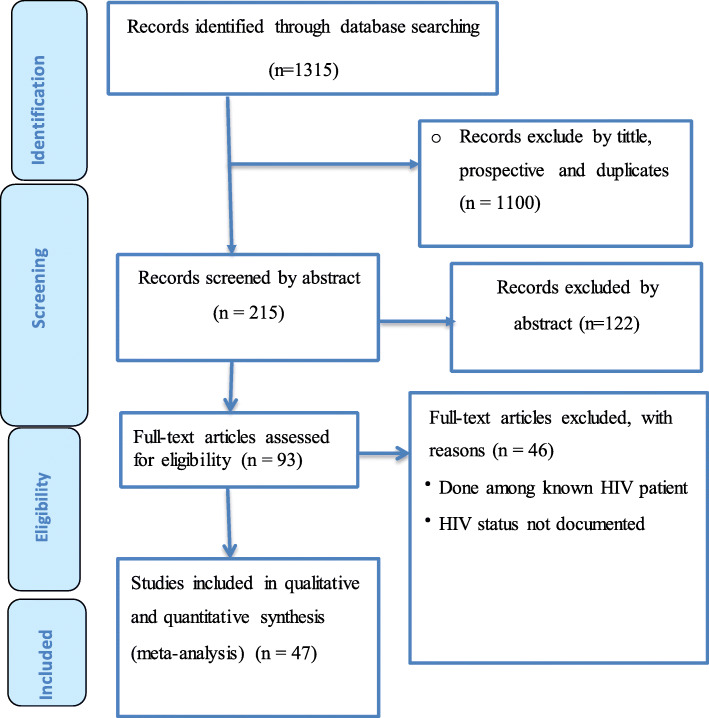
PRISMA flow chart of study selection

Overall, we retrieved 1315 potential articles using key terms and/or phrases. Of these, 93 full-text articles were reviewed in detail and the rest were excluded either due to duplicated title, or accessed only in the abstract. Here again, after a careful evaluation using inclusion criteria, we included only 47 studies for the final meta-analysis (Fig. 1).

### Characteristics of the included studies

A total of 347,896 TB patients were identified among the 47 included articles. The articles were published between 2010 to 2019 and undertaken between 2002 to 2017. Forty-two [21–26, 28–62] of studies used data collected before 2015 and remaining four studies [27, 63–66] from 2015 to 2017. The sample size of the studies varying from 162 [32] to 272,526 [45]. In this meta-analysis, eight of the nine regions and one of the two city administrative in Ethiopia represented. Sixteen of the studies were from Amhara region [31–34, 36, 39, 42, 44, 46, 48, 49, 51, 53, 57, 62, 65], eleven from Oromia region [23, 25, 28, 29, 41, 45, 49, 50, 59, 61, 66], six from Addis Ababa city [26, 27, 38, 40, 54, 63] and the rest from other regions. We identified three studies among children [26, 38, 56], and, three among adults [22, 32, 49], and the remaining 41 among all age groups (Table 1).

**Table 1:** Summary of studies assessing the proportion of unknown HIV status among patients with tuberculosis included in the analysis (*n* = 47)

Author, Year	Year Study	Study Region	Age Group	Health Facility	Study Design	Total TB	HIV R(%)	HIVNR (%)	HIV Unknown
Wondale et al 2017) [21]	2004–2014	SNNPR	All	Hospital	RC	2156	315 (14.60)	1289 (59.8)	552 (25.60)
Adane et al*,* 2018 [22]	2011–2015	Tigray	Adult	HC	RC	496	54 (11.00)	424 (85.00)	18 (3.63)
Ejeta et al., 2015 [23]	2009–2013	Oromia	All	HC	RC	1175	201 (17.10)	924 (78.60)	84 (7.15)
Getnet et al, 2017 [24]	2009–2014.	Somalia	All	Hospital	RC	1378	63 (4.60)	1311 (95.40)	4 (0.29)
Worku et al, 2018 [63]	2011–2016	Addis Ababa	All	Hospital	RC	340	58 (17.10)	263 (77.40)	19 (5.59)
Ramos et al, 2010 [25]	2002–2007	Oromia	All	Hospital	RC	2225	38 (1.70)	242 (10.90)	1943 (87.33)
Muluye et al, 2018 [66]	2012–2016	Oromia	All	Hospital	RC	995	68 (6.80)	889 (89.30)	38 (3.82)
Hailu et al,2014 [26]	2007–2011	Addis Ababa	Children	HC	RC	2708	346 (12.80)	945 (34.89)	1600 (59.08)
Arega et al., 2019 [27]	2015–2017	Addis Ababa	All	Hospital	RC	1876	117(6.70)	77 (4.10)	1682 (89.66)
Gebremariam et al.,2016 [28]	2008–2014	Oromia	All	HC	RC	1649	156 (10.00)	1406 (90.00)	87 (5.28)
Tafess et al, 2018 [29]	2004–2014	Oromia	All	Hospital	RC	1755	287 (16.40)	1174 (66.90)	294 (16.75)
Birlie et al, 2015 [30]	2008–2012	Amhara	all	HC	RC	810	141 (18.30)	631 (77.90)	38 (4.69)
Melese et al, 2018 [31]	2008–2013	Amhara	All	HC	RC	303	10 (3.30)	67 (22.10)	226 (74.59)
Berihun et al., 2018 [32]	2010–2015	Amhara	Adult	HC	RC	162	6 (3.70)	48 (29.60)	108 (66.67)
Endris et al., 2014 [33]	2007–2011	Amhara	All	HC	RC	417	24 (5.80)	182 (43.60)	211 (50.60)
Jaleta et al., 2017 [34]	2013–2015	Amhara	All	Hospital	RC	1820	315 (17.30)	621 (34.10)	884 (48.57)
Simieneh et al., 2017 [35]	2009–2014	SNNPR	All	Hospital	RC	1961	246 (13.9)	1519 (77.46)	196 (9.99)
Mekonnen et al., 2016 [36]	2011–2014	Amhara	All	HC	RC	990	227 (24.00)	704 (74.20)	18 (1.82)
Berhe et al., 2012 [37]	2009–2011	Tigray	All	HC	RC	470	35(8.60)	271 (66.60)	101 (21.49)
Tilahun et al., 2016 [38]	2009–2013	Addis Ababa	Children	HC	RC	491	82 (28.20)	209 (42.56)	200 (40.73)
Zenebe et al., 2016 [39]	2010–2015.	Amhara	All	Hospital	RC	1761	459 (26.06)	1235 (70.13)	67 (3.80)
Assefa et al., 2017 [40]	2013–2013	Addis Ababa	All	Hospital	RC	710	148 (20.84)	533 (30.26)	29 (4.08)
Sintayehu et al., 2014 [41]	2010–2013	Oromia	All	Hospital	RC	2043	328 (16.05)	1650 (80.76)	27 (1.32)
Tefera et al., 2016 [42]	2009–2013	Amhara	All	Hospital	RC	1280	262 (20.46)	645 (50.39)	373 (29.14)
Asebe et al., 2015 [43]	2011–2013	Gambella	All	Hospital	RC	1156	280 (24.22)	792 (60.05)	84 (7.27)
Kebede et al., 2017 [44]	2014–2015	Amhara	All	Hospital	RC	227	56 (24.60)	135 (59.50)	36 (15.86)
Sisaya et al., 2018 [45]	2009–2015	Oromia	All	Hospital	RC	272,526	13,680 (11.8)	101,588 (37.2)	157,258 (57.70)
Biruk et al., 2016 [46]	2008–2012	Amhara	All	Hospital	RC	1584	212 (13.40)	420 (26.50)	952 (60.10**)**
Yakob et al., 2018 [47]	2012–2015	SNNP	All	HC	RC	725	72 (9.90)	645 (91.10)	80 (11.03)
Addis et al., 2013 [48]	2008–2011	Amhara	All	HC	RC	482	123 (38.10)	200 (61.90)	159 (32.99)
Yadeta et al., 2013 [49]	2009–2011	Oromia	Adult	Hospital	RC	682	215 (32.20)	467 (68.47)	14 (2.05)
Akessa et al., 2015 [50]	2012–2013	Oromia	All	Hospital	RC	510	114 (22.40)	354 (69.40)	42 (8.24)
Yilma et al.,2019 [51]	2008–2014	Amhara	All	Hospital	RC	1952	600 (30.73)	1072 (54.92)	208 (10.66)
Ayele et al., 2015 [52]	2009–2014	SNNP	All	HC	RC	3722	499 (13.41)	3002 (80.66)	221 (5.94)
Woldeamanuel etal,2018 [53]	2013–2015	Amhara	All	Hospital	RC	262	114 (45.80)	135 (54.20)	13 (4.96)
Demile et al., 2018 [54]	2014–2015	Addis Ababa	All	Hospital	RC	389	34 (8.90)	321 (84.30)	26 (6.68)
Ejetab et al., 2018 [64]	2015–2017	Gambella	All	Hospital	RC	995	96 (9.64)	141 (14.18)	758 (76.18)
Zenebe et al.,2016 [55]	2010–2013	Afar	All	Hospital	RC	3634	510 (14.03)	1991 (54.79)	133 (3.66)
Asres et al., 2016 [56]	2008–2014	SNNPR	Children	HC	RC	790	77 (9.74)	535 (67.73)	178 (22.53)
Gebreegziabher et al.,2016 [57]	2007–2013	Amhara	All	HC	RC	15,140	716 (4.68)	6086 (40.19)	8338 (55.07)
Asebe et al.,2014 [58]	2003–2014	Gambella	All	Hospital	RC	4300	423 (9.83)	1021 (23.74)	2856 (66.42)
Abebe et al., 2015 [59]	2008–2012	Oromia	All	Hospital	RC	2107	178 (8.45)	1775 (84.24)	156 (7.40)
Alemu et al., 2017 [60]	2009–2014	Gambella	All	Hospital	RC	2519	605 (24.02)	1579 (62.68)	335 (13.30)
Tachbele et al., 2017 [61]	2007–2012	Oromia	All	Hospital	RC	916	158 (17.25)	553 (60.37)	2 05 (22.38)
Tarekegne et al., 2016 [62]	2009–2012	Amhara	All	Hospital	RC	2096	404 (19.27)	1601 (76.38)	91 (4.34)
Amante et al., 2015 [67]	2007–2012	Harar	All	HC	RC	976	179 (18.34)	460 (43.17)	261 (26.74)
Shibabaw et al., 2018 [65]	2011–2016	Amhara	All	Hospital	RC	235	61 (25.96)	162 (68.93)	12 (5.11)

*SNNPR* South national nationality and people of representative, *HIV* Human immune Virus, *TB* Tuberclosis, *R* Reactive, *NR* Non-Reactivity: tuberculosis, *RC* a retrospective cross-sectional study, *HC* health center, children age < 15 years

The majority (*n* = 38) of the studies conducted among different types of TB and only nine studies [27, 34, 37, 40, 44, 45, 54, 64, 65] among pulmonary TB (PTB). Based on the Acid Fast Staining (AFB), the proportion of AFB positive and AFB negative ranged from 9% [35] to 84% [26], and 8% [26] to 66% [60] respectively. The proportion of the EPTB varied from 8% [26] to 25% [60]. With regard to HIV infection status among TB patients, the HIV-positive ranged from 2% [25] to 44% [53], HIV-negative ranged from 4% [27] to 95% [24], and unknown HIV status ranged from 1% [24] to 90% [27].

### Quality of the included studies

We assessed the quality of all studies using the JBI Critical Appraisal Checklist for prevalence studies. Based on the assessment, all the included studies scored over 60% (6/9), and none of the included studies were deemed of poor quality and excluded (Additional file 1).

### Meta-analysis

#### Heterogeneity and publication bias

**Fig. 2:**
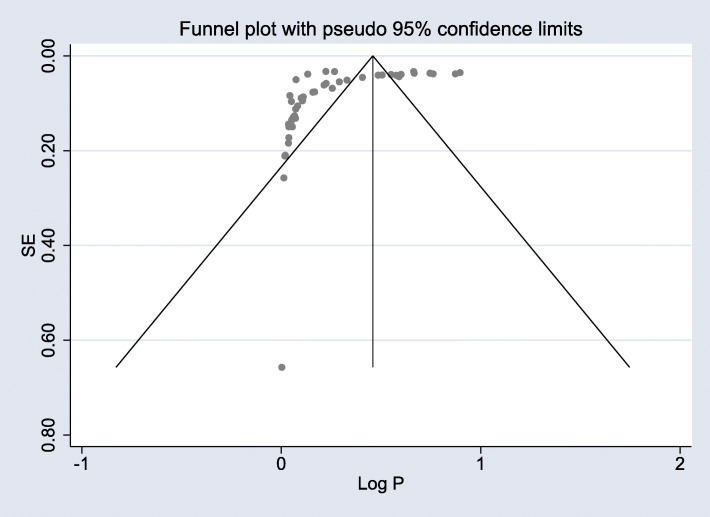
Funnel plot in which the vertical line provides an estimate of the effect size and the diagonal line shows the precision of individual studies with 95% confidence intervals

We assessed for heterogeneity and publication bias of 47 studies. The analysis showed substantial heterogeneity of the Q test (*p* < 0.001) and I^2^ statistics (I^2^ = 99.9%). A funnel plot for the publication bias was not symmetrical (Fig. 2). However, we did not find evidence of publication bias using Egger’s test (*P* = 0.94).

#### Unknown HIV status among tuberculosis patients

**Fig. 3:**
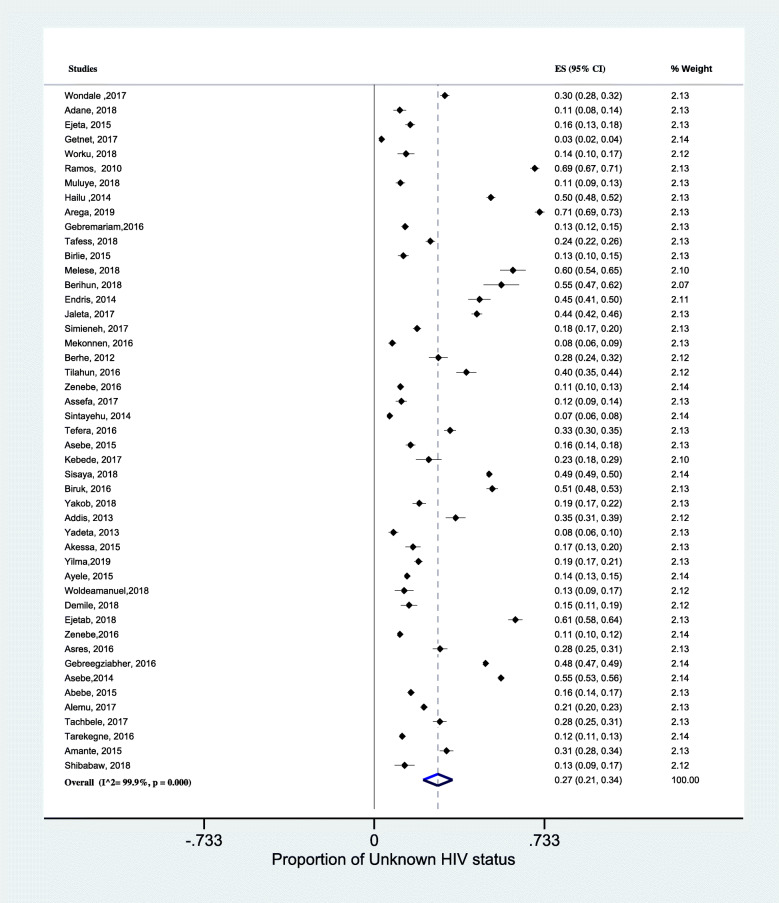
Forest plots of the pooled proportion of unknown HIV status among TB patients. *Legend:* SNNPR; South nation, nationality and people of representative

We presented the proportion of unknown HIV status among TB patients in a forest plot (Fig. 3). The overall pooled proportion of unknown HIV status from the random-effects model was 27% (95% CI; 21–34, I^2^ = 99.9%, *p* < 0.001) (about 73% had known status).

### Subgroup analysis

**Fig. 4:**
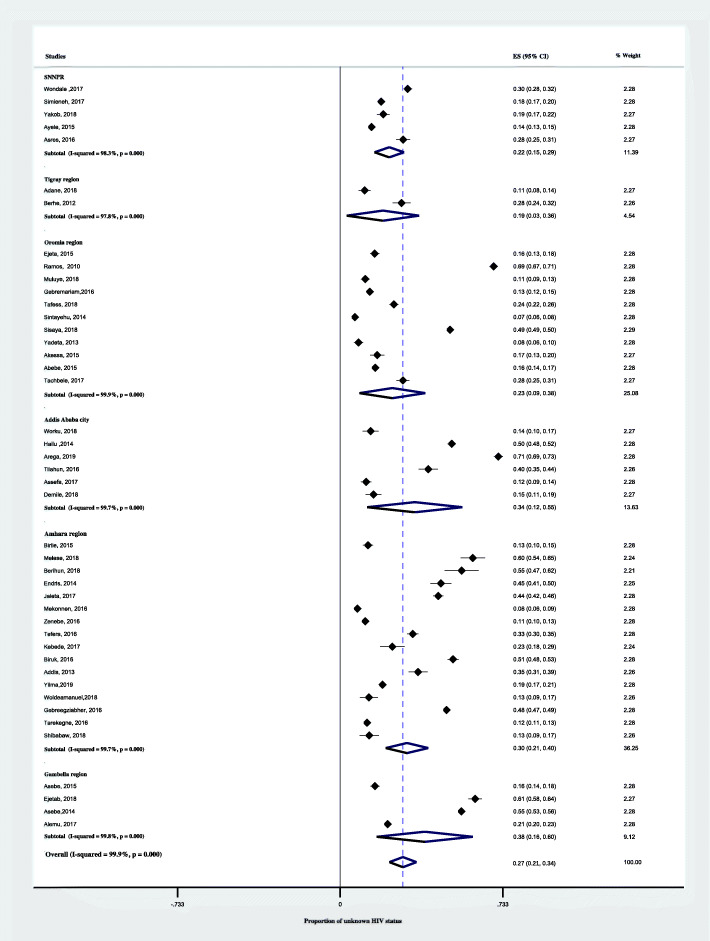
Forest plots showing subgroup analysis of the proportion of unknown HIV status among TB patients based on the region in Ethiopia

**Fig. 5:**
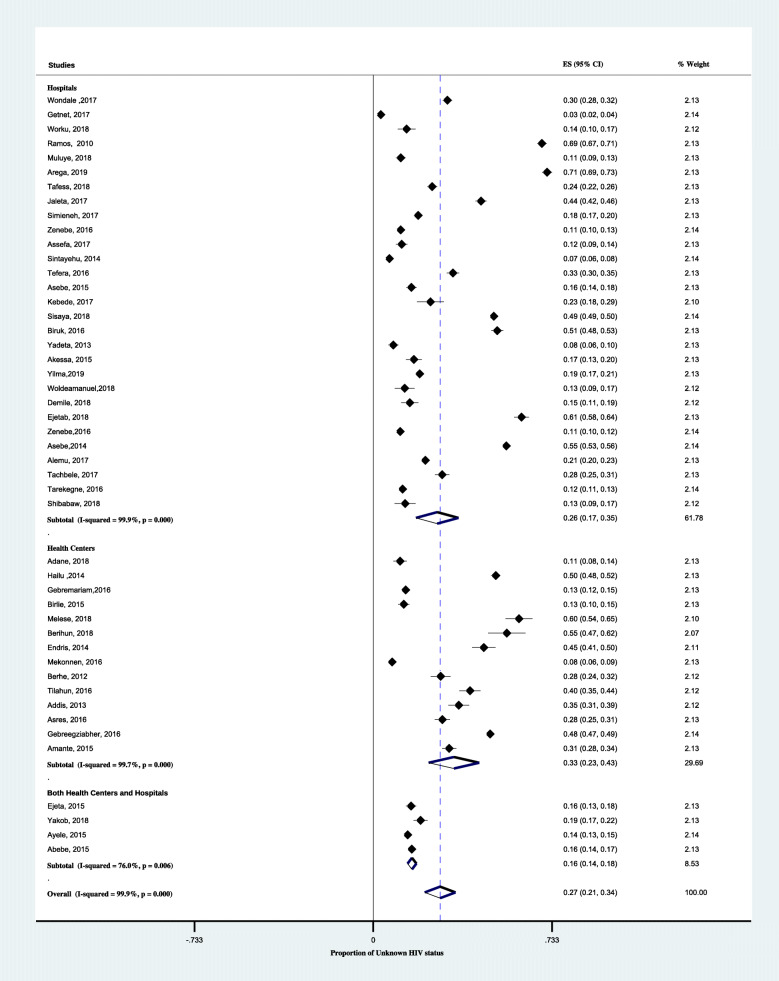
Forest plots showing subgroup analysis of the proportion of unknown HIV status among TB patients based on the study facility in Ethiopia

**Fig. 6:**
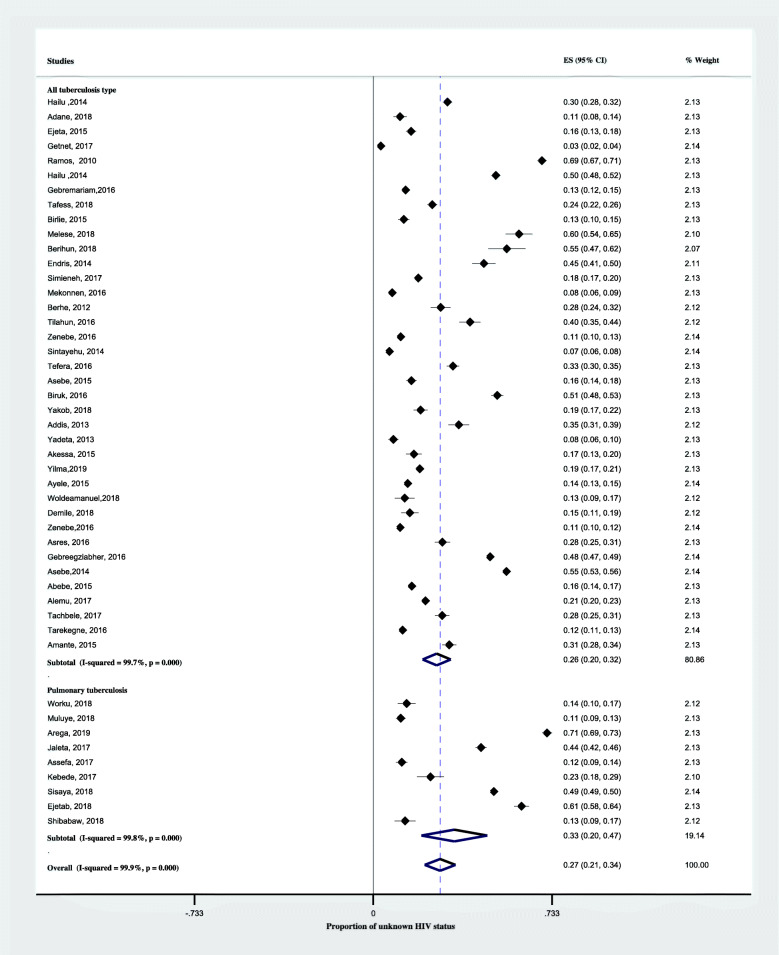
Forest plots showing subgroup analysis of the proportion of unknown HIV status among TB patients based on types of tuberculosis in Ethiopia

**Fig. 7:**
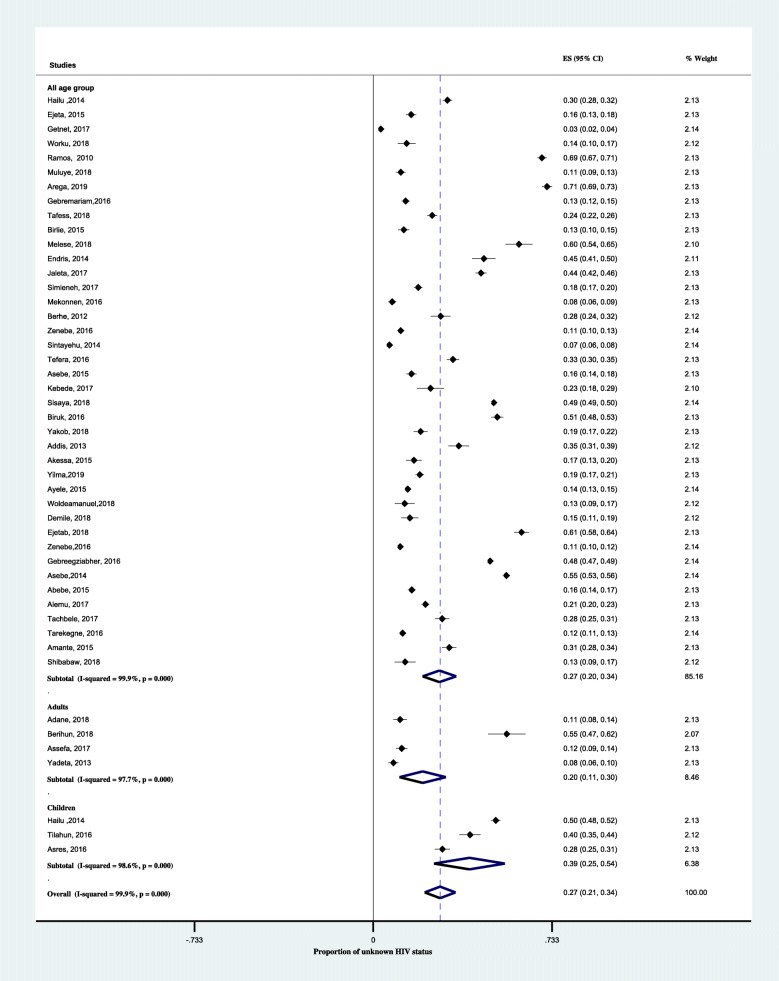
Forest plots showing subgroup analysis of the proportion of unknown HIV status among TB patients based on the age groups in Ethiopia

In a subgroup analysis by region, the pooled proportion of unknown HIV status was highest in Gambella, 38% (95% CI; 16–60%) followed by Addis Ababa, 34% (95% CI; 12–55%) and Amahara region, 30% (95% CI; 21–40). As shown in Fig. 4, the lowest pooled proportion Unknown HIV status was in Tigray region, 19% (95% CI;3–38). Another subgroup analysis by setting, the pooled proportion of unknown HIV status was 33% (95% CI; 23–43%) in the health centers alone and 26% (95% CI;17–35%) in hospitals (Fig. 5). According to the anatomical classification of TB, the pooled proportion of unknown HIV status was 33% (95% CI; 20–47) among pulmonary tuberculosis and 26% (95% CI; 20–32) among all types of TB patients (Fig. 6). The age-based analysis showed that the pooled proportion of unknown HIV status was 39% (95% CI; 25–54) and 20% (95% CI; 11–30) among children (< 15 years) and adults (Fig. 7).

### Sensitivity analysis

The sensitivity analysis revealed that our findings were robust and not dependent on a single study. The pooled estimated prevalence varied between 26% (20–34%) and 28% (21–34%) after a single study deleted (See Additional files 2).

## Discussion

As WHO recommended, routine testing of tuberculosis patients for HIV is crucial in HIV epidemic countries like Ethiopia. This is crucial to monitor and assess the HIV/TB collaborative prevention and care programs [6]. In this review, we found that the pooled proportion of unknown HIV status among TB patients was 27%. This is higher than the national HIV/TB surveillance (including 79 senile sites) that reported 89% of TB patients were screened for HIV [68]. In this surveillance, the investigators trained the data collectors, they prepared standardized data collection guidelines and supervised the data collection process. This might be the reason for the lower level of unknown HIV status among tuberculosis patients in national surveillance, unlike our findings. Another meta-analysis of 13 studies in Ethiopia, amid to assess the prevalence of HIV among TB patients, reported a lower prevalence rate (6.4%) of unknown HIV status compared to our findings [69]. The differencemight be related to the number of studies included or because they included prospective studies in their review. In cases, a study in Malawi showed that the routine HIV testing among TB patients was above 90% under research conditions, but lower (59%) with routine care conditions [70]. Ethiopia adopted the global 90–90-90 targets (2018–2020) with 90% of people with HIV know their status [71], but the current pooled analysis showed that there is major gap in achieving this target. The problem might be because of a weak health care system or lack of education and health service support, poor access, or lack of availability of screening materials.

In the subgroup analysis, we identified a high proportion of unknown HIV status in Gambella (38%) and Addis Ababa city (34%) compared to the other regions included in the reviews. The three (2005, 2011, and 2016) Ethiopian demographic health surveys reported the highest prevalence rate of HIV in Gambella and Addis Ababa [72]. Thus, the findings of this review showed a higher number of HIV-positive patients that were missed in these regions.

The pooled proportion of unknown HIV status is higher in health centres (33%) than in hospitals (26%). In Ethiopia, AFB-negative TB patients with typical clinical symptoms who do not respond to a trial antibiotic treatment and all AFB–Positive TB patients are treated with anti-TB at health centers. However, critically ill TB patients, TB patients with TB treatment history or presumptive drug-resistant TB, and EPTB patients are referred to the next level health facilities (primary, general, or tertiary hospital). Here, the patients are further investigated for comorbid conditions including HIV. This might be the potential reason for lower level of unknown HIV status in hospitals compared to health centers. The variation across the health settings for the same program might also relate to lack of counselling and testing service, poor behavioural change, or poor integration of the program in to the health system.

In this review, unknown HIV status among children (< 15 years) was higher than adults (39% vs. 20%). Similarly, HIV testing among children versus adults with TB in Vietnam revealed that about 70% of children had incomplete HIV test documentation [73]. In our country, testing children for HIV, including TB patients, is focused on the HIV serostatus of parents. This approach may miss other than parent to child (vertical) HIV transmission in adolescent age groups.

This is the first review aimed to determine the proportion of unknown HIV status among TB patients since the national HIV/TB collaborative activities initiated. All the studies included in the review fulfilled more than half of the JBI quality assessment check criteria, with no study excluded because of poor quality scores. As the HIV pandemic continues to fuel the global tuberculosis epidemic, including in Ethiopia, such review is significant to provide comprehensive data about HIV status among patients with TB and shows priority study area to investigate potential sources for the spread of HIV to the general population.

This study has certain limitations. As all the studies included were retrospective, this may overestimate the pooled proportion of unknown HIV status among tuberculosis patients. We detected high levels of heterogeneity across all the analyses and readers should interpret the pooled analysis and subgroup with caution.

## Conclusion

In this review about one-third of TB patients had unknown HIV status in Ethiopia. This showed a gap to achieve the national 90–90-90 HIV/AIDS strategic plan, which is expected to be achieved by 2020 and eliminate the HIV epidemic by 2030. Therefore, it is essential to strengthen TB/HIV collaborative program in Ethiopia in order to reduce the potential source for the ongoing HIV transmission. Additional researches, mainly longitudinal studies, should be a key priority to investigate factors for the unknown HIV status and the variations across the health settings, regions, types of tuberculosis,and age groups.

## Supplementary information

**Additional file 1.** Quality of studies included in the meta analysis tassese the proportion of unknown HIV status among patients with tuberculosis

**Additional file 2.** Sensitivity analysis of the proportion of unknown HIV status among patients with tuberculosis

## Data Availability

All the data and materials are available and available to any researcher wishing to use them.

## Abbreviations

CI: Confidence interval
HIV: Human immunodeficiency virus
TB: Tuberculosis
WHO: World Health Organization?
