# Supplementary material for: Unknown HIV status and the TB/HIV collaborative control program in Ethiopia: systematic review and meta-analysis
Source: BMC Public Health. 2020 Jun 29;20:1021. doi: 10.1186/s12889-020-09117-2 (PMC7325261; doi:10.1186/s12889-020-09117-2)
Supplement: Supplementary file 1 — Additional file 1. Quality of studies included in the meta analysis tassese the proportion of unknown HIV status among patients with tuberculosis [file 12889_2020_9117_MOESM1_ESM.docx]

| **Author** | **Q1** | **Q2** | **Q3** | **Q4** | **Q5** | **Q6** | **Q7** | **Q8** | **Q9** | **Score** |
| --- | --- | --- | --- | --- | --- | --- | --- | --- | --- | --- |
| Wondale et al 2017)[19] | Y | Y | Y | Y | NA | Y | UN | Y | NA | 6 |
| Adane et al, 2018 [20] | Y | Y | Y | Y | NA | Y | UN | Y | NA | 6 |
| Ejeta et al , 2015[21] | Y | Y | Y | Y | NA | Y | Y | Y | NA | 7 |
| Getnet et al, 2017[22] | Y | Y | Y | Y | NA | Y | Y | Y | NA | 7 |
| Worku et al, 2018[23] | Y | Y | Y | Y | NA | Y | UN | Y | NA | 6 |
| Ramos et al, 2010[24] | Y | Y | Y | Y | NA | Y | Y | Y | NA | 7 |
| Muluye et al, 2018[25] | Y | Y | Y | Y | NA | Y | Y | Y | NA | 7 |
| Hailu et al,2014 [26] | Y | Y | Y | Y | NA | Y | Y | Y | NA | 7 |
| Arega et al, 2019[27] | Y | Y | Y | Y | NA | Y | UN | Y | NA | 6 |
| Gebremariam et al,2016[28] | Y | Y | Y | Y | NA | Y | UN | Y | NA | 6 |
| Tafess et al, 2018 [29] | Y | Y | Y | Y | NA | Y | Y | Y | NA | 7 |
| Birlie et al, 2015 [30] | Y | Y | Y | Y | NA | Y | Y | Y | NA | 7 |
| Melese et al, 2018 [31] | Y | Y | Y | Y | NA | Y | Y | Y | NA | 7 |
| Berihun et al, 2018 [32] | Y | Y | N | Y | NA | Y | Y | Y | NA | 6 |
| Endris et al, 2014 [33] | Y | Y | Y | Y | NA | Y | UN | Y | NA | 6 |
| Jaleta et al, 2017 [34] | Y | Y | Y | Y | NA | Y | UN | Y | NA | 6 |
| Simieneh et al, 2017[35] | Y | Y | Y | Y | NA | Y | UN | Y | NA | 6 |
| Mekonnen et al, 2016[36] | Y | Y | Y | Y | NA | Y | UN | Y | NA | 6 |
| Berhe et al, 2012 [37] | Y | Y | Y | Y | NA | Y | Y | Y | NA | 7 |
| Tilahun et al, 2016[38] | Y | Y | Y | Y | NA | Y | UN | Y | NA | 6 |
| Zenebe et al, 2016[39] | Y | Y | Y | Y | NA | Y | Y | Y | NA | 7 |
| Assefa et al, 2017 [40] | Y | Y | Y | Y | NA | Y | Y | Y | NA | 7 |
| Sintayehu et al, 2014[41] | Y | Y | Y | Y | NA | Y | Y | Y | NA | 7 |
| Tefera et al, 2016 [42] | Y | Y | Y | Y | NA | Y | Y | Y | NA | 7 |
| Asebe et al, 2015[43] | Y | Y | Y | Y | NA | Y | UN | Y | NA | 6 |
| Kebede et al, 2017 [44] | Y | Y | Y | Y | NA | Y | Y | Y | NA | 7 |
| Sisaya et al, 2018 [45] | Y | Y | Y | Y | NA | Y | Y | Y | NA | 7 |
| Biruk et al, 2016 [46] | Y | Y | Y | Y | NA | Y | Y | Y | NA | **7** |
| Yakob et al, 2018 [47] | Y | Y | Y | Y | NA | Y | Y | Y | NA | 7 |
| Addis et al, 2013 [48] | Y | Y | Y | Y | NA | Y | U | Y | NA | 6 |
| Yadeta et al, 2013[49) | Y | Y | Y | Y | NA | Y | Y | Y | NA | 7 |
| Akessa et al, 2015 [50] | Y | Y | Y | Y | NA | Y | Y | Y | NA | 7 |
| Yilma et al,2019 [51] | Y | Y | Y | Y | NA | Y | Y | Y | NA | 7 |
| Ayele et al, 2015 [52] | Y | Y | Y | Y | NA | Y | Y | Y | NA | 7 |
| Woldeamanuel etal,2018[53] | Y | Y | Y | Y | NA | Y | Y | Y | NA | 7 |
| Demile et al, 2018 [54] | Y | Y | N | Y | NA | Y | Y | Y | NA | 7 |
| Ejetab et al, 2018 [55] | Y | Y | Y | Y | NA | Y | Y | Y | NA | 7 |
| Zenebe et al,2016 [56] | Y | Y | Y | Y | NA | Y | UN | Y | NA | 6 |
| Asres et al, 2016 [57] | Y | Y | Y | Y | NA | Y | Y | Y | NA | 7 |
| Gebreegziabher et al, 2016[58] | Y | Y | Y | Y | NA | Y | Y | Y | NA | 7 |
| Asebe et al,2014 [59] | Y | Y | Y | Y | NA | Y | Y | Y | NA | 7 |
| Abebe et al, 2015 [60] | Y | Y | Y | Y | NA | Y | Y | Y | NA | 7 |
| Alemu et al, 2017 [61] | Y | Y | Y | Y | NA | Y | Y | Y | NA | 7 |
| Tachbele et al, 2017 [62] | Y | Y | Y | Y | NA | Y | UN | Y | NA | 6 |
| Tarekegne et al, 2016 [63] | Y | Y | Y | Y | NA | Y | Y | Y | NA | 7 |
| Amante et al, 2015[64] | Y | Y | Y | Y | NA | Y | Y | Y | NA | 7 |
| Shibabaw et al, 2018[65] | Y | Y | N | Y | NA | Y | Y | Y | NA | 6 |

Y, Yes ,N,No , UN, unclear , NA,not applicable , Qs: the JBI appraisal tool for prevalence studies (2017)

**Additional file 1**.Quality of studies included in the meta analysis tassese the proportion of unknown HIV status among patients with tuberculosis
