# Supplementary material for: Unknown HIV status and the TB/HIV collaborative control program in Ethiopia: systematic review and meta-analysis
Source: BMC Public Health. 2020 Jun 29;20:1021. doi: 10.1186/s12889-020-09117-2 (PMC7325261; doi:10.1186/s12889-020-09117-2)
Supplement: Supplementary file 2 — Additional file 2. Sensitivity analysis of the proportion of unknown HIV status among patients with tuberculosis [file 12889_2020_9117_MOESM2_ESM.docx]

**Additional file** 2: Sensitivity analysis of the proportion of unknown HIV status among patients with tuberculosis

| **Study excluded** | **Proportion (95%CI)** |
| --- | --- |
| Wondale ,2017 | 27% (21-34) |
| Adane, 2018 | 28 (21-34) |
| Ejeta, 2015 | 28 ( 21-34) |
| Getnet, 2017 | 28 (22-34) |
| Worku, 2018 | 28( 21-34) |
| Ramos, 2010 | 26( 20-34) |
| Muluye, 2018 | 28 (21-34) |
| Hailu ,2014 | 27 (20-34) |
| Arega, 2019 | 26 ( 20-34) |
| Gebremariam,2016 | 28 (21-34) |
| Tafess, 2018 | 27 (21-34) |
| Birlie, 2015 | 28 ( 21-34) |
| Melese, 2018 | 27 ( 20-34) |
| Berihun, 2018 | 27 ( 20-34) |
| Endris, 2014 | 27 (21-34) |
| Jaleta, 2017 | 27 (21-34) |
| Simieneh, 2017 | 27 (21-34) |
| Mekonnen, 2016 | 28 (22-34) |
| Berhe, 2012 | 27( 21-34) |
| Tilahun, 2016 | 27( 21-34) |
| Zenebe, 2016 | 28 (21-34) |
| Assefa, 2017 | 28 (21-34) |
| Sintayehu, 2014 | 28 (22-34) |
| Tefera, 2016 | 27 ( 21-34) |
| Asebe, 2015 | 28 (21-34) |
| Kebede, 2017 | 27 ( 21-34) |
| Sisaya, 2018 | 27 ( 21-34) |
| Biruk, 2016 | 27 (20-34) |
| Yakob, 2018 | 27 ( 21-34) |
| Addis, 2013 | 27( 21-34) |
| Yadeta, 2013 | 28 (21-34) |
| Akessa, 2015 | 27 (21-34) |
| Yilma,2019 | 27 ( 21-34) |
| Ayele, 2015 | 28 (21-34) |
| Woldeamanuel,2018 | 28 ( 21-34) |
| Demile, 2018 | 28 ( 21-34) |
| Ejetab, 2018 | 27 (20-34) |
| Zenebe,2016 | 28( 21-34) |
| Asres, 2016 | 27 (21-34) |
| Gebreegziabher, 2016 | 27 (20-34) |
| Asebe,2014 | 27 (20-34) |
| Abebe, 2015 | 28 (213-34) |
| Alemu, 2017 | 27 ( 21-34) |
| Tachbele, 2017 | 27 (21-34) |
| Tarekegne, 2016 | 28 (21-34) |
| Amante, 2015 | 27 (21-34) |
| Shibabaw, 2018 | 28 (21-34) |

Key. The analysis is based on the random effects model
